# Acute seizures and status epilepticus in immune effector cell associated neurotoxicity syndrome (ICANS)

**DOI:** 10.1038/s41408-022-00657-x

**Published:** 2022-04-13

**Authors:** Jacqui-Lyn Saw, M. Hasib Sidiqi, Michael Ruff, Sara Hocker, Hassan Alkhateeb, Stephen M. Ansell, N. Nora Bennani, David Dingli, Suzanne R. Hayman, Patrick B. Johnston, Prashant Kapoor, Saad J. Kenderian, Taxiarchis V. Kourelis, Shaji K. Kumar, Jonas Paludo, Mithun V. Shah, Mustaqeem A. Siddiqui, Rahma Warsame, Allison Rosenthal, Marie Grill, Januario E. Castro, Jason Siegel, Zaid H. Abdel Rahman, Mohamed A. Kharfan-Dabaja, Elson So, Yi Lin

**Affiliations:** 1 Department of Neurology, Mayo Clinic, Rochester, MN USA; 2 Department of Hematology, Mayo Clinic, Rochester, MN USA; 3 Department of Hematology, Mayo Clinic, Phoenix, AZ USA; 4 Department of Neurology, Mayo Clinic, Phoenix, AZ USA; 5 Department of Neurology, Mayo Clinic, Jacksonville, FL USA; 6 Division of Hematology-Oncology, Mayo Clinic, Jacksonville, FL USA

**Keywords:** Immunotherapy, Neurological disorders, Non-hodgkin lymphoma, Cancer immunotherapy

**Dear Editor,**

Chimeric Antigen Receptor (CAR) T cell therapy is a transformative treatment that is being increasingly utilized. The novel mechanism of action lends itself to unique toxic profiles. These toxicities manifest in two forms: cytokine release syndrome (CRS) and immune effector cell associated neurotoxicity syndrome (ICANS). ICANS typically manifests as a toxic encephalopathy with a complex delirium. Seizures have been reported as rare occurrences, with very little detail regarding the clinical presentation, assessment or management [1–5].

The electroencephalogram (EEG) is a critical diagnostic test for the assessment of seizures and nonconvulsive status epilepticus (NCSE). In ICANS, the indications and utility of the EEG remain poorly defined [1]. EEG findings have been reported anecdotally and in small case series [1, 6]. The most common finding has been that of non-specific generalized slowing and generalized periodic discharges [6]. EEG is crucial for the diagnosis of episodic seizures and NCSE in ICANS, and it may assist in confirming and assessing the degree of encephalopathy. We present a uniform cohort of relapsed/refractory B cell lymphoma patients treated with a single FDA-approved CD-19 targeting CAR-T, axicabtagene ciloleucel (Yescarta).

We conducted a retrospective review of all patients with B-cell non-Hodgkin lymphoma who received Yescarta through either clinical trial or standard of care at the Mayo Clinic in Minnesota, Arizona and Florida between June 2016 and October 2018. EEG recordings were independently reviewed in accordance with the American Clinical Neurophysiology Society’s Standardized Critical Care EEG Terminology [7]. The clinical course and grade of ICANS were determined using the National Cancer Institute Common Terminology Criteria for Adverse Events (CTCAE) system [8]. Lee’s modified criterion was used for CRS grading [9]. Patients with encephalopathy attributed to other causes were excluded. The study was approved by the Mayo Clinic Institutional Review Board. Statistical analysis was performed on JMP software (SAS, Cary, NC). Patient and disease-related factors between groups were compared using the χ2 test for categorical variables, and the Wilcoxon signed rank test for continuous variables.

A total of 33 patients received 33 doses of CAR-T cell therapy (axicabtagene ciloleucel) during the study period. Neurologic symptoms attributable to CAR-T, grade I-IV based on CTCAE were identified in 21 (64**%**) patients, 13 of whom underwent evaluation with EEG (long-term video monitoring and routine EEGs). Nine patients (27%) in the whole cohort had grade ≥3 ICANS. Patient demographics for those presenting with ICANS are shown in Table 1a.

**Table 1:** a Characteristics of patients. b Summary of EEG Findings.

Variable	Whole cohort (*n* = 33)	ICANS cohort (*n* = 21)	EEG (*n* = 13)	No EEG (*n* = 8)	*P* value
**a**					
Age, median (range), years	52 (26–65)	52 (26–65)	52 (26–65)	54 (38–64)	0.74
Male, n (%)	27 (82)	18 (86)	11 (85)	7 (88)	>0.99
Diagnosis, n (%)					
DLBCL	26 (79)				
T/F Follicular	5 (15)				
PMBCL	2 (6)				
Number prior therapies, median (range)		4 (2–8)	4 (2–8)	4 (2–7)	>0.99
Prior ASCT, n (%)	13 (39)	8 (38)	3 (23)	5 (63)	0.16
Prior CNS involvement, n (%)	4 (12)				
Days to CRS, median (range)		1 (0–5)	1 (0–5)	2.5 (0–5)	0.19
Duration CRS, median days (range)		6 (1–15)	5 (1–15)	7 (1–10)	0.38
CRS Grade n (%)					0.43
I		3 (14)	1 (8)	2 (25)	
II		17 (81)	11 (45)	6 (75)	
III		1 (5)	1 (8)	0	
Days to ICANS, median (range)		5 (0–19)	4 (0–8)	5.5 (3–19)	0.11
Duration ICANS, median days (range)		6 (1–14)	6 (1–14)	4.5 (1–12)	0.42
Neurologic symptoms grading by CTCAE n (%)					0.14
I		6 (29)	2 (15)	4 (50)	
II		6 (29)	3 (23)	3 (38)	
III		7 (33)	6 (46)	1 (12)	
IV		2 (9)	2 (15)	0	
Hospital admission, median days (range)		13 (6–55)	14 (8–55)	13 (6–15)	0.51
Features of ICANS n (%)					
Somnolence		8 (30)	8 (62)	0 (0)	
Focal neurological deficit*		4 (15)	4 (30)	0 (0)	
Global Aphasia		4 (15)	4 (30)	0 (0)	
Treatment					
Tocilizumab		12 (57)	10 (77)	2 (25)	0.03
Dexamethasone		16 (76)	12 (92)	4 (50)	0.048
ASM escalation (increased dose and/or addition of alternate ASM)		11	10	1	
Surgical intervention		1	1 (0.03)	0 (0)	

	Neurologic symptoms grade <3 (*n* = 4)	Neurologic symptoms grade >3 (*n* = 8)
**b**		
Type of EEG		
Routine	3	0
Prolonged	2	8
EEG Parameters		
Normal background	1	0
Focal slowing	1	2
Generalised non-specific slowing	4	7
Generalised periodic discharges (<2Hz)	1	4
Generalised periodic discharges (>2.5Hz)	0	3*
Generalised rhythmic delta with evolution	0	1^#^

*ASCT* autologous stem cell transplant, *CRS* cytokine release syndrome, *ASM* antiseizure medications, *DLBCL* diffuse large B cell lymphoma, *T/F* transformed, *PMBCL* primary mediastinal large B cell lymphoma, *ASCT* autologous stem cell transplant, *CNS* central nervous system, *EEG* electroencephalogram; *nonconvulsive status epilepticus; ^#^electrographic seizure episodes.

All patients with ICANS had a preceding diagnosis of CRS. The observed rates of ICANS in the standard of care population treated at our centers are comparable to the incidence reported in the published literature [10]. Age, gender, time to CRS, duration of CRS and grade of CRS were not statistically different when comparing the EEG cohort to those without EEG. There was no statistical difference in the severity and the median duration of neurotoxicity symptoms between the EEG cohort and the no EEG cohort, however this was likely representative of sample size as a clinical difference did exist (≥Grade 3 neurotoxicity: 61% EEG cohort vs 12% No EEG cohort, *p* = 0.07; median duration of ICANS: 6 days EEG cohort vs 4.5 days No EEG cohort, *p* = 0.42).

All patients who had ICANS had some form of language disturbance ranging from mild word finding difficulty to global aphasia. Only 4 (19%) patients had focal neurological signs excluding dysphasia. One patient had left-sided neglect, one had dysphagia, one had-right sided weakness and the final patient had “higher cognitive function impairment”. None of these four patients had NCSE at the time of their EEG monitoring. Eight patients (38%) had somnolence as a key clinical feature of their ICANS. Four (19%) patients had profound global aphasia, as opposed to mild word finding difficulty. One patient was suspected to have clinical seizure activity, but EEG was not pursued. Four patients in the cohort had previous CNS lymphoma, with no active disease at the time of treatment and did not have seizures. Importantly, all patients were on an antiseizure medication (ASM) at baseline.

Six patients had lumbar puncture performed. Two patients had elevated CSF opening pressure (>20 cm H_2_O). One patient required a ventriculostomy with an extra-ventricular drainage device, as well as treatment with tocilizumab and steroids. The other was placed on mannitol, as well as tocilizumab and steroids with symptomatic improvement.

Thirteen patients received EEG with video recordings (2 routine and 11 prolonged inpatient monitoring studies). The EEGs were independently reviewed by two authors (Dr So and Dr Saw) according to the ACNS guidelines. The main findings are summarized in Table 1b. The severity of EEG findings reflected the clinical severity of the neurotoxicity. All EEGs except one (92%) were abnormal. The normal EEG was in a patient with low grade neurotoxicity (<Grade 3). On the other hand, all patients with high grade neurotoxicity (≥Grade 3) had EEG abnormalities. The most common EEG abnormality was generalized non-specific slowing in 11 patients, followed by generalized periodic discharges in 8 patients (Fig. 1a). The rate of the generalized periodic discharges in 3 patients was 2.5 Hz or faster, which indicates NCSE. All three patients had high grade neurotoxicity. A fourth patient with high grade neurotoxicity had EEG seizures with episodic rhythmic delta slowing that evolved in amplitude, morphology and rhythmicity and distribution (Fig. 1b).

**Fig. 1:**
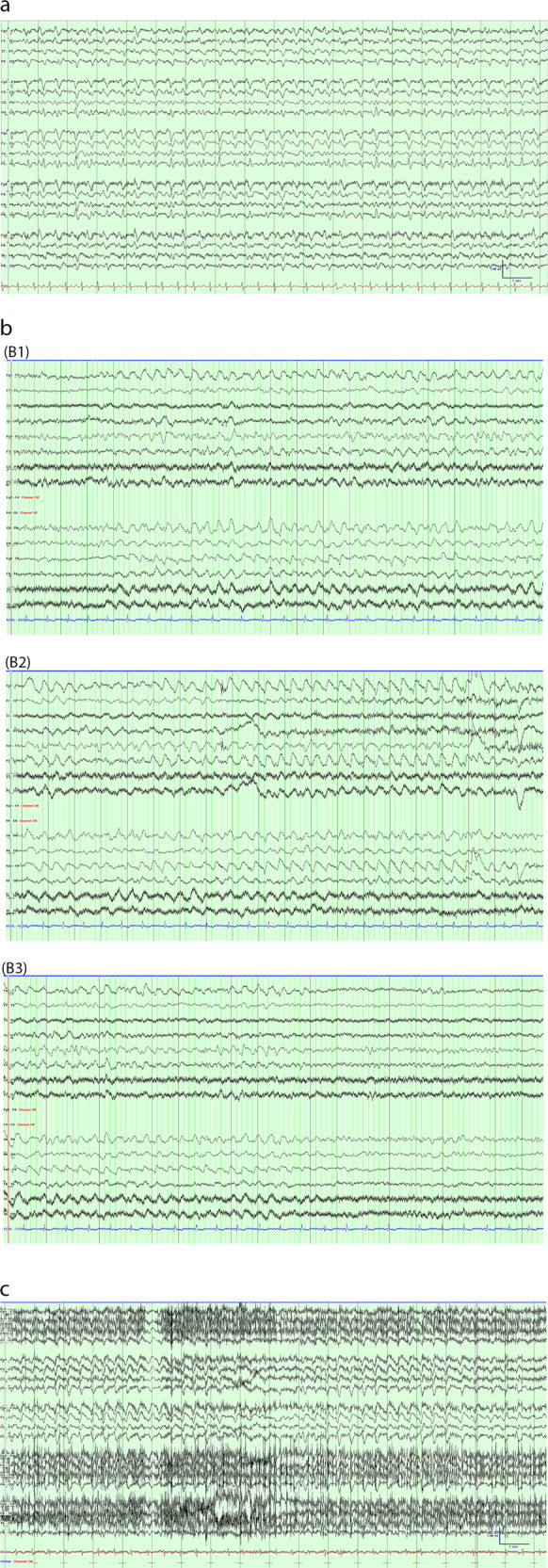
A-C EEG examples. **a** Generalized periodic discharges (2.5–3 Hz); (**b**): Samples of EEG seizure activity at onset (B1), during seizure (B2), and at offset (B3), showing rhythmic slowing that evolved in amplitude, morphology rhythmicity, and distribution; (**c**): EEG during episode of >2.5 Hz generalized periodic discharges associated with intermittent rhythmic clonic twitching involving the right abdomen and upper limb.

Of the three patients with NCSE, clinical manifestations of seizures were minimal in two and absent in one. A 48 yo male on day 4 post infusion developed a non-fluent dysphasia, and was administered one dose of tocilizumab and dexamethasone. Prolonged EEG monitoring was initiated. On day 6 after infusion, the patient was obtunded with intermittent rhythmic clonic twitching involving the right abdomen and upper limb with associated >2.5 Hz generalized periodic discharges (Fig. 1c). The patient with episodic EEG seizures had no clinical correlates with the episodes. All four patients had EEG seizures even when receiving antiseizure drugs, until the doses were escalated or additional medications were given. All patients were given prophylaxis with levetiracetam 500 mg twice daily, which was escalated to 1000 mg twice daily with ICANS onset. Additional anti-seizure medications were added in 3 patients as follows: one case had lacosamide added, another had phenobarbitone and midazolam added, and the remaining case had additional lorazepam. Following resolution of ICANS, the ASM was tapered off. If patients did not develop ICANS the ASM was tapered off by one month post discharge. If residual symptoms of ICANs were present at one month post CAR T cell therapy, they were then seen by a Neuro-Oncology specialist who provides recommendations regarding taper. EEG seizures were not observed in those with low neurotoxicity scores. All seizure patients were discharged from hospital with no evidence of encephalopathy or seizures. Two of these patients had progression of their lymphoma and they subsequently died. The remainder had no evidence of epilepsy at last follow-up.

The EEG pattern of generalized periodic discharges occurred in seven of our eight ICANs patients with high grade neurotoxicity, and in only one of 5 patients with low grade neurotoxicity (Table 1a). The dual significance of generalized periodic discharges are: (1) the appearance in the setting of acute and diffuse structural, toxic or metabolic brain injury; (2) the high association with either clinical or subclinical seizure occurrence, or both. The occurrence of these discharges at 2.5 Hz or faster for at least 20% of the recording met the criteria [11] of NCSE in three of our patients with high grade neurotoxiciy. Current evidence describes a strong association of IFN gamma, GM-CSF, IL-6, IL-10, IL-15, and possibly other inflammatory signalling molecules with ICANS [12]. Gust et al suggest examining other disorders associated with GPDs with triphasic morphology and cerebral oedema [12]. These have been described with hepatic encephalopathy and hyperammonemia. Proposed common mechanisms in pathophysiology include the dysfunction of glial water handling at the neurovascular unit, excitotoxicity, and energy failure [11]. In rats, cerebral oedema from hepatotoxicity is related to upregulation of IL-6, IL1B and TNF [13]. Whilst these cytokines are related and not definitively causative, further understanding of their role may augment future treatments.

Prompt recognition of ICANS is critical to optimizing management of patients, and it largely relies on the provider’s clinical assessment. Seizures or seizure like-activities have been listed as clinical manifestations of ICANS, with the reported incidence of seizure activity varying depending on CAR-T cell product type [14]. However, there is little description of the frequency and characteristics of seizures in ICANS. A more recent series reported a single patient from a total of 100 who had seizure activity [15]. In contrast to other cohorts, our study reveals 50% of the high grade ICANS patients were experiencing seizures, with the majority being in NCSE. Of note, a significant proportion of our patients did not receive EEG, and presumably some of these patients may have had self-limiting NCSE. The higher rates of seizure activity in our cohort may reflect the unique toxic phenotype of the product. This is an important observation, and we suspect that individual CAR-T products have unique profiles with respect to ICANS-related complications.

The diagnosis of NCSE can be challenging as very recurrent or continuous EEG seizures occur with only subtle or no clinical symptoms. EEG is required to establish the diagnosis, because encephalopathic symptoms such as coma or mental confusion in critically ill patients do not by themselves distinguish patients with NCSE from patients without NCSE. On the other hand, NCSE itself may cause encephalopathy including coma. Other symptoms if present are very subtle, such as small random ocular or facial muscle twitches [15]. Even more visible clinical seizure activity may underrepresent and underestimate the burden of ongoing EEG seizures. Prolonged nonconvulsive seizures in the primate animal model result in ischemic neuronal injury in the cortex, thalamus and hippocampus [16]. In the non-CAR-T cell population, mortality in critically ill patients is increased with greater burden of nonconvulsive seizures and status epilepticus [17]. Prophylactic ASM in patients receiving CAR-T cells remains controversial and its use typically varies according to the type of CAR-T product and/or institutional guidelines [1]. Therefore, EEG recording should be performed when ICAN symptoms appear, especially in those with high grade neurotoxicity. Initiation of ASM or steroids, or both, is effective in resolving both seizures and other ICAN symptoms, as was the case with our cohort.

As CAR-T usage expands, prompt recognition, treatment and understanding of seizures in ICANs will be of increasing importance. Optimal management of ICANS remains unclear. Steroids have been found to mitigate symptoms of encephalopathy in some patients. Given the potential suppressive effect of steroid on CAR-T cells, sparing use of toxicity management to minimize the risk of compromising treatment effect has been the driving principle. Infection prophylaxis is also important in the care of these patients.

The use of EEG in ICANS has been variable and knowledge of its utility has been limited due to lack of standardized practice and rigorous analysis. We suspect that the rate of NCSE is under-reported due to this, yet its identification is crucial to the management of ICANS. The rates of NCSE and seizures may be underestimated if the EEG is underutilized. We wish to highlight the EEG as a diagnostic tool to identify this important manifestation of ICANs.

## Reporting summary

Further information on research design is available in the Nature Research Reporting Summary linked to this article.

## Supplementary information

Reporting Summary
